# The Impact of Pulsatile Flow on Suspension Force for Hydrodynamically Levitated Blood Pump

**DOI:** 10.1155/2019/8065920

**Published:** 2019-06-03

**Authors:** Yang Fu, Yimin Hu, Feng Huang, Maoying Zhou

**Affiliations:** ^1^School of Mechanical & Energy Engineering, Zhejiang University of Science & Technology, Hangzhou, China; ^2^State Key Laboratory of Fluid Power & Mechatronic Systems, Zhejiang University, Hangzhou, China; ^3^College of Metrology & Measurement Engineering, China Jiliang University, Hangzhou, China; ^4^School of Mechanical Engineering, Hangzhou Dianzi University, Hangzhou, China

## Abstract

Hydrodynamically levitated rotary blood pumps (RBPs) with noncontact bearing are effective to enhance the blood compatibility. The spiral groove bearing (SGB) is one of the key components which offer the suspension force to the RBP. Current studies focus on the suspension performance of the SGB under continuous flow condition. However, the RBP shows pulsatile characteristics in the actual clinical application, which may affect the suspension performance of the SGB. In this paper, the impact of pulsatile flow upon the suspension force from the SGB is studied. A model of the SGB with a groove formed of wedge-shaped spirals is built. Then, the CFD calculation of the hydrodynamic force offered by designed SGB under simulated pulsatile flow is introduced to obtain the pulsatile performance of the suspension force. The proposed method was validated by experiments measuring the hydrodynamic force with different bearing gaps. The results show that the suspension force of the SGB under pulsate flow is the same as under steady flow with equivalent effective pressure. This paper provides a method for suspension performance test of the SGB.

## 1. Introduction

Implanted rotary blood pumps (RBPs) are considered as replacement therapy for heart disease due to the lack of organ donation. The RBP requires a rotor bearing to maintain longtime operation. Compared with contact bearings, noncontact bearings avoid wearing and thrombus formation around the bearing [1]. Magnetic bearings have been applied in RBPs, enhancing the blood compatibility of the RBPs [2]. However, magnetic bearings need sensors and control system to guarantee the suspension, which increases the complexity of the implanted device. Thus, hydrodynamic bearings, which offer suspension force depending on its hydraulic characteristics, were widely studied for RBPs [3–5].

Spiral groove bearing (SGB), which is small in size and has excellent loading capacity, is considered as a good structure used for the hydrodynamic bearing. The SGB was first introduced by Kink and Reul in the RBP to minimize the size of the implant device, and the mock-loop test result [6] showed that the hydraulic performance of the RBP was not influenced compared to the contact bearing. Yamane et al. [7] made a new groove shape for the SGB to levitate the impellers of RBP, and thrombus formation was reduced in their design. Experimental studies by Zhu et al. [8] pointed out that the pressure generated by SGBs is sufficient to support the rotor. Han et al. [9, 10] developed a novel SGB that the groove width decreases with increasing spiral radius to improve washout of the RBP. The exposure time of blood flow to high shear stresses of the RBP is demanded to be lower in order to improve its hemocompatibility. Amaral et al. [11] introduced an optimized SGB design which can enhance the washout flow between the rotor and pump casing, significantly decreasing the exposure time and improving the overall efficiency of the pump.

The existing studies of the SGBs are mainly focused on the suspension performance under continuous flow condition, where the RBPs are tested with given constant flow rate and the outlet pressure of zero. However, in the actual clinical application, the inlet pressure and outlet pressure of the RBPs vary according to the periodic pressure change of the ventricular, as the RBPs are connected between the left ventricular and aorta [12–14]. Therefore, the flow of the RBPs shows pulsatile characteristics even with fixed rotating speed [12, 13]. Studies [15–17] show that the pulsatile characteristics of the RBPs affect the performance of the RBPs, such as hydraulic characteristics, blood compatibility, etc. However, no relevant studies were reported about the impact of the RBPs' pulsatile characteristics on the suspension performance of the SGBs.

For the conventional SGB with logarithmic spiral grooves, mathematical analysis [18] has been deduced with the conclusion that the hydrodynamic force on the bearing was not affected by the pressure variation. As mentioned in [9], the RBP used novel SGBs with the groove formed of wedge-shaped spirals has superior load/flow characteristics, but it is difficult to construct CFD model of the RBP for analysis of its suspension performance. Thus, computational fluid dynamics (CFD) [19] analyses and experiments were conducted in this article to investigate the behavior of SGBs under physiological pulse conditions. The remainder of this article offers the following:

1. CFD model of the SGB with wedge-shaped spiral grooves is built. Then, the CFD calculation of the hydrodynamic force offered by designed SGB under simulated pulsatile flow is introduced to obtain the pulsatile performance of the suspension force.
2. The proposed method is validated by comparing experimentally measured suspension force with CFD calculation under different bearing gaps.

The impact of pulse flow on the suspension performance of the SGB to the RBP has been studied in this paper, which provides a method for suspension performance test of the SGB. The CFD calculation and experiment test have been implemented. Also, the results show that the suspension force of the SGB under pulsate flow is the same as under steady flow with equivalent effective pressure.

## 2. Materials and Methods

### 2.1. Structure of SGB

**Figure 1:**
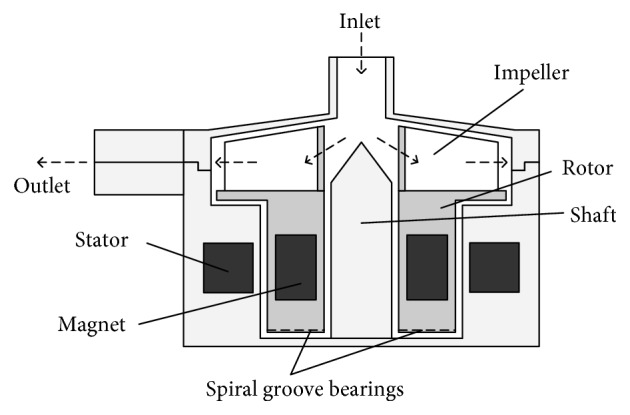
The schematic of the RGB.

The structure of the RBP with hydrodynamic bearings is shown in Figure 1. The pump consists of the pump housing, impeller, stator, and shaft. The SGB is fixed on the rotor which offers suspension force to levitate the rotor, whose performance will be discussed. There is a big difference in size between the pump and the groove, where the size order of the groove depth and the bearing gap is 10^−5^ m and the pump as 10^−2^ m. As mentioned in the method part, more than 1,800,000 elements are used to fully represent the computational domain of the SGB. Correspondingly, the number of grids will be very huge to cover the whole blood pump, which will exceed the capability of general computers. Thus, only SGB is modeled and studied in this paper.

**Figure 2:**
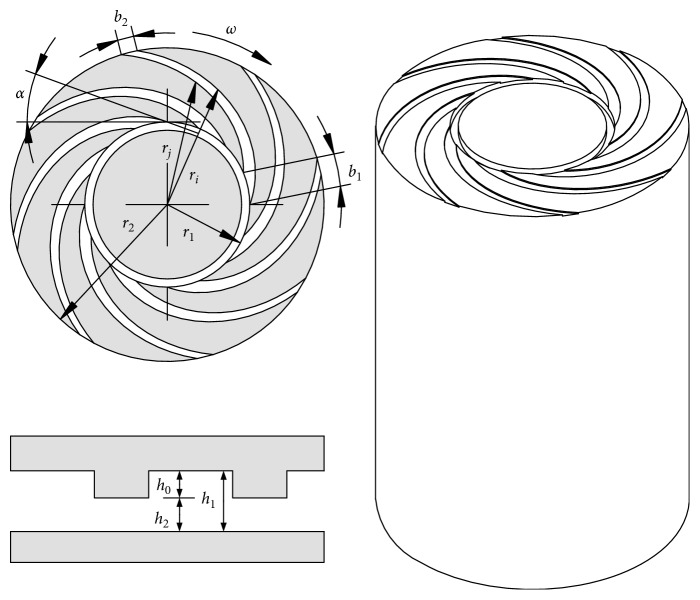
The axial and cross-sectional view of a SGB.

The wedge-shaped SGB is based on the design of Han et al. [9], where the groove width decreases along the flow path. Thus, the relative motion between the SGB and the corresponding surface facilitates the flow through the grooves. The purpose of the novel SGB structure is to maintain high load-carrying capacity while producing a large flow rate to reduce blood damage. The two groove profiles in polar coordinates are expressed as

(1) $$\begin{array}{c} r_{i}=r_{1}e^{\theta_{i}\tan\;\alpha },\\ r_{j}=r_{1}e^{\left(\theta_{j}-\left(b\left(r_{i}\right)/r_{i}\right)\right)\tan\;\alpha }, \end{array}$$

where *r* is the radius, *b* is the groove width, *θ* is the phase angle, *α* is the spiral angle and remains constant, *r*_1_ is the radius at zero polar angle, and *b*_1_ is the groove width at *r*_1_. And the groove width is a function of *r*:

(2) $$\begin{array}{c} b\left(r\right)=\frac{b_{2}-b_{1}}{r_{2}-r_{1}}\left(r-r_{1}\right)+b_{1}, \end{array}$$

where *b*_1_=*b*(*r*_1_), *b*_2_=*b*(*r*_2_), and *b*_1_ > *b*_2_. Thus, the groove width decreases along the radius. The geometry of a SGB is shown in Figure 2. The groove depth and bearing gap are *h*_0_ and *h*_2_, respectively. The total distance between the groove and the corresponding surface is *h*_1_=*h*_0_+*h*_2_.

### 2.2. Numerical Analysis

Numerical simulation was carried out to calculate the suspension force of the SGB under continuous and pulsatile flow condition. According to Han's study [9], the geometrical parameters of the SGB used in the simulation are listed in Table 1, with groove depth and the spiral angle of 80 *μ*m and 20°. Also, the number of grooves was set to 8.

**Figure 3:**
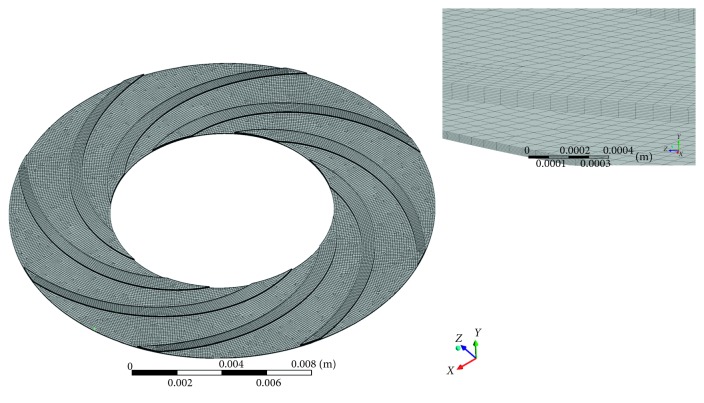
CFD mesh result of the SGB.

As shown in Figure 3, three-dimensional grids of the flow field were developed with ANSYS ICEM CFD. Prism grids are generated using the same settings for all cases, and the boundary-layer grids include more than 5 layers of elements. The total number of elements was between 1,800,000 and 3,000,000 according to the actual fluid domain. A mesh refinement study was conducted for 50,000 up to 10,000,000 elements. The estimated hydrodynamic force showed a deviation lower than 5% for meshes over 1,700,000 elements. The computation was conducted through ANSYS CFX and assumed to achieve convergence when the RMS (root mean square) residuals were below 1*e*^−5^. In this paper, simulations were carried under continuous flow and pulsatile flow as follows.

First, steady-state simulations were performed to acquire the suspension forced by the SGB. In the normal operation of the SGB in the RBP, the bearing gap is commonly between 20 *μ*m to 300 *μ*m [7, 11, 20]. Therefore, the bearing gap in the simulation was chosen from 20 *μ*m to 300 *μ*m, and the data were calculated every 20 *μ*m. The boundary condition was set with inlet and outlet pressure of zero. The estimated hydrodynamic force was further compared with the experiments.

**Figure 4:**
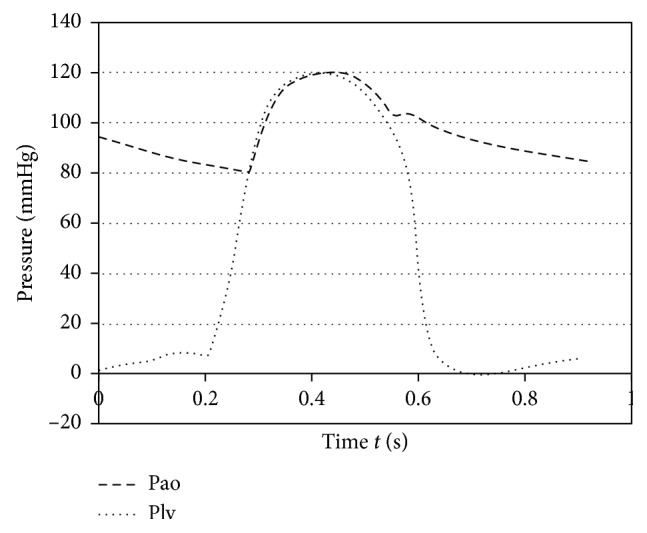
Left ventricular pressure (Plv) and aorta pressure (Pao) with the cardiovascular model in healthy conditions [21].

Second, numerical simulation was operated under pulsatile flow condition to figure out whether the suspension force of the SGB was affected. Shi and Korakianitis [21] built a numerical model of the cardiovascular system, where the left ventricular pressure (Plv) and the aortic pressure (Pao) in healthy condition were given as shown in Figure 4. The left ventricular pressure varies between 10 and 120 mmHg, and the aortic pressure changes from 80 to 120 mmHg. In the actual operation, the inlet and outlet of the RBP were connected to the ventricular and aorta, respectively. Therefore, the pressure at *r*_1_ and *r*_2_ was set equal to Plv and Pao to simulate the pulsate flow condition, which was close to the actual operation condition of the SGB. For comparison, a series of steady-state simulations were carried out with time invariant boundary conditions based on Pao-Plv at a certain time, as shown in Table 2.

### 2.3. Experimental Validation

**Figure 5:**
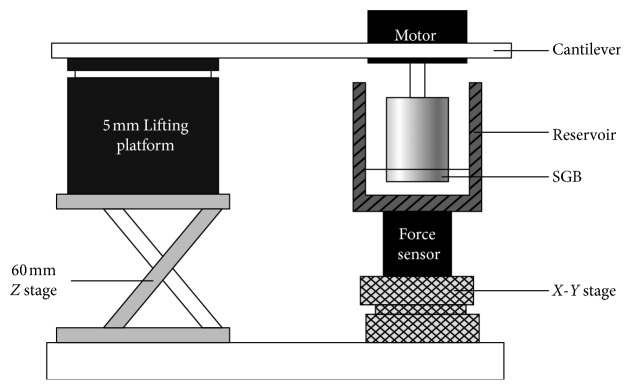
Test system for the measurement of hydrodynamic force.

Experiments were carried out to validate the suspension performance test method and the effectiveness of the simulation presented above. The schematic of the experimental setup is shown in Figure 5, which is modified from previous devices on suspension force test for hydrodynamically levitated blood pump reported in colleagues' research [11, 22]. A SGB manufactured with ABS was fixed on a motor shaft (EC32 Flat, maxon motor ag, Sachseln, Switzerland) which was controlled by a monitor (maxon motor ag, Sachseln, Switzerland). The motor was supported by a 5 mm lifting platform (Winner Optics, WN01VM5) with 5 *μ*m resolution through a cantilever made of aluminum alloy. The 5 mm lifting platform was mounted on a 60 mm *Z* stage (Winner Optics, WN08VM60) that regulates height roughly. The SGB to be tested was immerged in a cylindrical reservoir, and the fluid was a mixture of water and glycerol with 38% glycerol in weight. A 6-DOF force and moment sensor (ATI Nano 43) was fixed between the reservoir and a *X*-*Y* stage (Winner Optics, WN202WM25M) to adjust the SGB to the center of the reservoir. All signals were acquired by a DAQ card (National Instruments, Austin, Texas, USA), and a control program was written with LabVIEW (National Instruments, Austin, Texas, USA) to monitor the motor speed and the sensor response.

In this experiment, the hydrodynamic force on the counter plane of the SGB with different bearing gaps changed by the elevator-platform was measured. According to Newton's third law, the suspension force offered by the SGB was equal to the hydrodynamic force on the counter plane. The geometrical parameters of the SGB were designed the same to the simulation. The rotating speed of the motor was fixed at 3000 rpm, the same as the specified speed of the blood pump. The bearing gap ranged from 80 *μ*m to 300 *μ*m in 20 *μ*m steps.

### 2.4. Validation of the Model Accuracy

**Figure 6:**
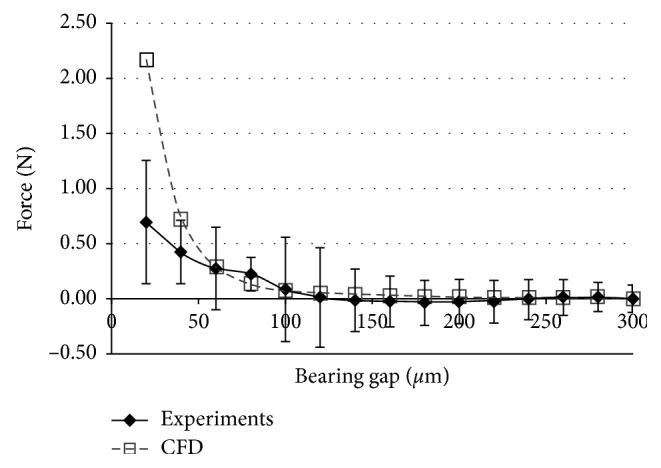
Comparison between experiment and simulation results under steady state.

The suspension force of the SGB measured by the experiment was compared with the CFD calculated result to validate the effectiveness of the CFD model, as shown in Figure 6. In the experiment and CFD calculation, the pressure of the inlet and outlet was set to zero.

The suspension force offered by SGB decreases with the increase of the bearing gap. It was found that the simulation results matched the experiment results well especially when the bearing gap was larger than a critical value, which was about 60 *μ*m in this design. When the bearing gap was small, it was found that the suspension force of the simulation was larger than the experimental value. As shown in Figure 6, the suspension force showed a large discrepancy with the variation of the bearing gap when the bearing gap was below 50 *μ*m. In the experiment, the bearing gap was regulated by the displacement platform, leading to the difficulty in adjusting the bearing gap to a precision level, especially in the tiny distance range. Therefore, the simulation result was different from the experiment when the bearing gap was small. In general, the experiment result fits the simulation. Thus, the conclusion could be made that the numerical simulation model built above was valid to estimate the suspension forced by the SGB.

### 2.5. Impact of Pulsatile Flow on the Hydrodynamic Force

**Figure 7:**
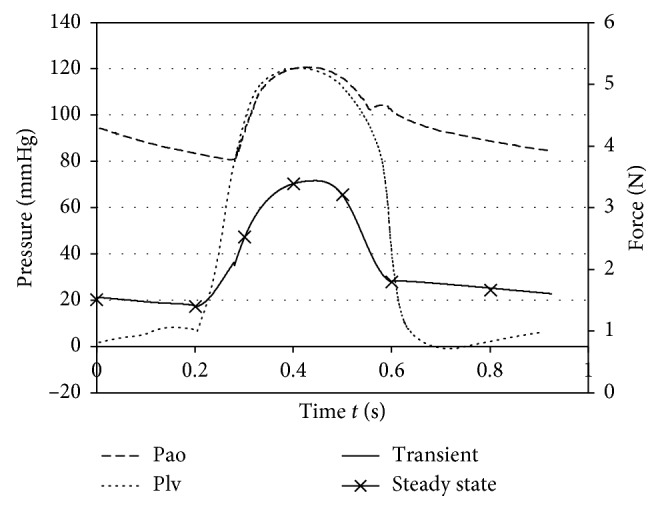
Comparison between transient and steady-state results by CFD calculation.

The hydrodynamic force under pulsatile flow was calculated by CFD, where the pressure at inlet and outlet of the SGB was set equal to Plv and Pao, as shown in Figure 7. First, the transient simulation was carried out with the pressure varied continuously according to the Plv and Pao, and the hydrodynamic force was calculated continuously. Then, CFD calculation was carried out under steady state with several given pressures the same as the transient condition as comparisons. It can be found that the hydrodynamic force under pulsatile flow was the same as steady state value. In this calculation, the hydrodynamic force was calculated including the pressure on the surface of the SGB. Thus, the hydrodynamic force was relative to the set pressure on inlet and outlet. Through comparison between the transient results and steady state results, it can be concluded that the suspension force of the SGB under pulsating flow is the same as under steady flow with equivalent effective pressure.

## 3. Conclusion

The impact of the pulsatile flow on the suspension force of the SGB was studied in this paper. A model of the SGB with a groove formed of wedge-shaped spirals was built. CFD calculation of the designed SGB was carried out to obtain the effects of the suspension force according to the changing flow. The hydrodynamic force with different bearing gaps was measured by experiment. The measured suspension forces were in accord with the CFD calculation results under steady state, which validated the effectiveness of CFD calculation for the SGB. Also, the CFD calculation results showed that the suspension force of the SGB under pulsating flow is the same as under steady flow with equivalent effective pressure. In this study, the simulation and experiments were implemented for the SGB, not including the whole region of the RBP. The motion of the pump rotor is affected by the coupling of the suspension force of the SGB, the normal force of the impeller, and the magnetic force of the motor. In future works, the influence of the latter two forces will be considered and studied.

## Figures and Tables

**Table 1:** Geometrical parameters of tested SGB.

Inner radius *r*_1_	5 mm
Outer radius *r*_2_	9.5 mm
Groove angle *α*	20°
Groove width *b*_1_/*b*_2_	3.92 mm/1.96 mm
Groove depth *h*_0_	80 *μ*m
Number of grooves *k*	8

**Table 2:** Boundary conditions for different pressure heads.

Time (s)	Pressure at *r*_1_ (mmHg)	Pressure at *r*_2_ (mmHg)
0.0	1.2	94.3
0.2	7.2	83.5
0.4	120.3	120.1
0.6	39.6	102.1
0.8	2.3	88.8

## Data Availability

The data used to support the findings of this study are available from the corresponding author upon request.
